# NAMazing: Déjà Vu at the lab bench - Why animal-free science is the new automobile

**DOI:** 10.1016/j.namjnl.2025.100022

**Published:** 2025-04-30

**Authors:** Thomas Hartung

**Affiliations:** a Johns Hopkins University, Center for Alternatives to Animal Testing (CAAT), USA; b University of Konstanz, CAAT-Europe, Germany

The ongoing technological shift in life sciences—from traditional animal testing to microphysiological systems (MPS) and artificial intelligence (AI)—remarkably mirrors the historic transition from horse-drawn carriages to automobiles over a century ago. History may not repeat itself exactly, but it certainly rhymes, particularly when groundbreaking innovations challenge established norms.

In the early 1900s, automobiles were frequently dismissed as noisy, dangerous, and unreliable novelties. Critics, confident in their skepticism, infamously mocked broken-down cars by shouting, “*Get a horse!*” One prominent editorial from the period earnestly argued, "*The horse is here to stay, but the automobile is only a novelty—a passing fad.*" Others asserted that automobiles would never match the dependability, convenience, or emotional connection provided by horses. These today almost humorous yet at the time sincere remarks, once passionately argued, have become historical punchlines (Fig. 1, Fig. 2), yet their echoes are strikingly audible in today’s resistance toward innovative biomedical technologies such as MPS and AI for regulatory decision making.

**Fig. 1:**
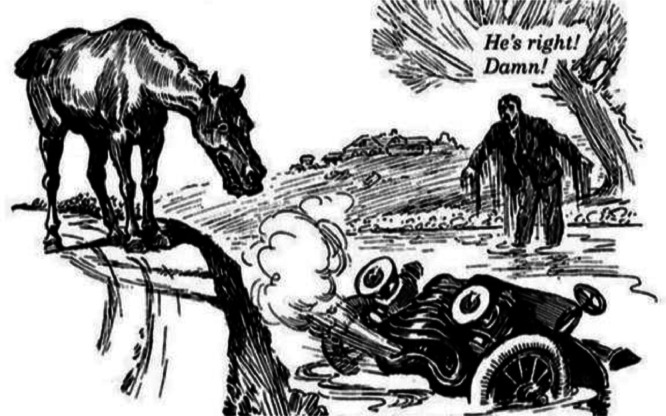
Historical cartoon.

source:https://www.linkedin.com/pulse/horse-vs-automobile-현상-sean-yim-임-jisfc/

**Fig. 2:**
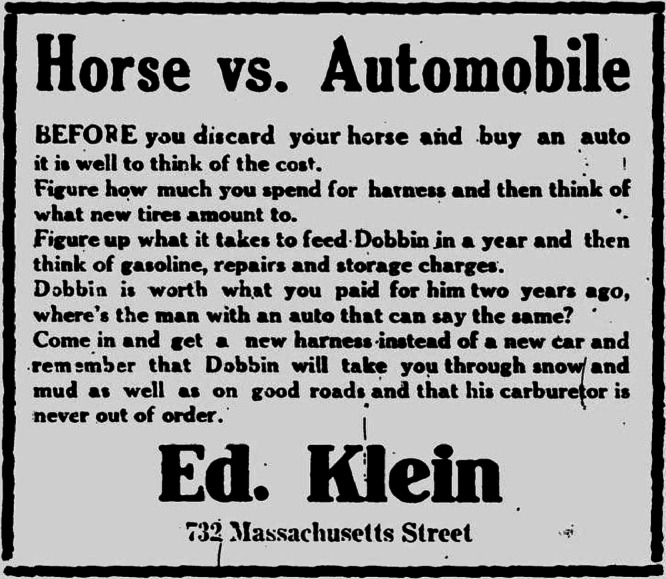
Historical advertisment.

Source:https://www.linkedin.com/pulse/horse-vs-automobile-현상-sean-yim-임-jisfc/

Just as the automobile required entirely new infrastructures—paved roads, gas stations, standardized traffic rules, and maintenance facilities—the widespread adoption of MPS and AI likewise necessitates extensive new frameworks (Hartung and Smirnova, 2025; Hartung et al., 2025). Transitioning to automobiles was successful precisely because infrastructure investments progressed alongside technological innovations. Similarly, today's shift toward MPS (Marx et al., 2016, 2020, 2025) and AI demands (Hartung, 2023; Kleinstreuer and Hartung, 2024) investments in specialized laboratories, regulatory frameworks, quality assurance (Pamies et al., 2020, 2022, 2024), digital databases, and computational capabilities. The historic push for paved roads was mirrored today by calls for standardization in laboratory practices and harmonization of regulatory protocols and reporting standards (Hartung et al., 2019) to accommodate these revolutionary biomedical methods (Hartung and Tsatsakis, 2021).

Moreover, during the automotive revolution, public safety became a primary concern, fueled by sensational media reports highlighting the dangers of automobile accidents. Fearful citizens hesitated to trust these new machines, much as current audiences question the reliability and transparency of MPS and AI. Early automotive safety initiatives, such as later mandatory seat belts and speed limits, were pivotal in building public trust. Today, similar transparent validation frameworks (Hartung 2024b; Hartung et al., 2024a,b; Hartung and Kleinstreuer, 2025) are being championed in collaboration with agencies such as the FDA and EPA to foster public and scientific confidence. Former FDA Commissioner Robert Califf emphasized, "*We must develop adaptable regulation to keep pace with rapid advancements in AI*", a sentiment echoing early regulatory efforts that sought to balance innovation with safety during the automotive transition.

Educational initiatives were fundamental in easing society’s shift from horses to cars. Early automobile adopters had to be instructed not only in driving but also in basic vehicle maintenance, safety protocols, and navigation. Similarly, today's revolutionary biomedical technologies demand extensive educational efforts to familiarize researchers, regulators, and the public with their operation and benefits. However, the user interfaces of AI are getting easier every day. Modern cell culture is supported by a supply chain, which assures quality. And consultancies and Contract Research Organizations do the job for you, if you desire so. Organizations like the Global Coalition for Regulatory Science Research (GCRSR) actively engage stakeholders through educational frameworks such as the TREAT principle—Trustworthiness, Reproducibility, Explainability, Applicability, and Transparency—highlighting the importance of clear, consistent communication to foster trust (Hartung et al., 2025) (Fig. 3).

**Fig. 3:**
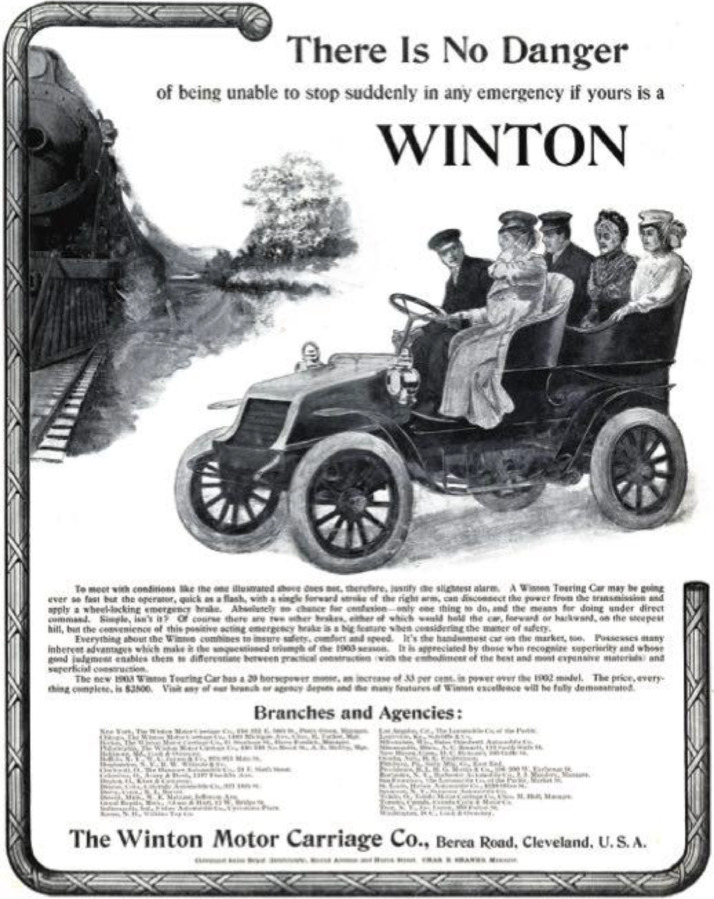
“Risk communication”.

Winton, January 24, 1903, available at: https://www.saturdayeveningpost.com/2017/01/get-horse-americas-skepticism-toward-first-automobiles/

Today's animal-free technologies, notably "organs-on-chips," promise to replicate human biology with unprecedented precision (Hartung and Smirnova, 2025; Marx et al. 2025), much as automobiles promised unprecedented mobility and speed. Unlike animal testing limited by inherent interspecies differences, MPS employs human cells within carefully engineered environments designed to replicate human physiological conditions closely. Coupled with AI-driven analysis (Smirnova et al., 2018), these technologies offer superior predictive capabilities, efficiency, and scalability, ultimately promising safer and more relevant biomedical outcomes.

Nevertheless, just as early automobiles faced significant technological and regulatory hurdles, today’s MPS and AI methodologies encounter considerable validation challenges. We have championed a vision for next-generation validation known as "e-validation." (T Hartung et al., 2024b; Hartung and Kleinstreuer, 2025). This approach utilizes AI algorithms to optimize chemical selection, simulate validation scenarios, and continuously monitor method performance. Such forward-thinking validation methods parallel historical shifts in maintenance from repairing horse harnesses to diagnosing automotive engines—both initially mysterious processes that required new knowledge, specialized training, and innovative tools.

The regulatory landscape is also evolving, reflecting historical parallels. The recent strategic announcements from the FDA^1^ and EPA^2^ constitute a landmark shift (Hansell and Hartung, 2025) akin to historical regulatory acceptance of automobiles. EPA's groundbreaking decision to phase out animal testing entirely represents a significant regulatory endorsement of innovative, animal-free technologies, much as historical laws supported the automobile industry’s expansion by establishing standards for vehicle safety and reliability. Similarly, the FDA’s recent roadmap lays clear principles for integrating AI into regulatory processes, emphasizing global collaboration, transparency, and continuous validation. These strategic initiatives echo early automotive regulatory responses, aiming to harness technological potential safely and effectively.

As we navigate from horses to chips, it is instructive—and amusing—to recall historical skepticism. Cartoons and satirical editorials from the early 20th century often ridiculed car enthusiasts, portraying them as misguided dreamers. Skeptics claimed automobiles were impractical, overly complex, and fundamentally flawed—criticisms now humorously antiquated. Today’s debates about MPS and AI echo these historical jibes, with critics questioning whether technology could ever reliably replace traditional animal models.

Yet, just as the early pioneers persevered, today's scientific community must also champion innovation in the face of skepticism. The successes and eventual ubiquity of automobiles should inspire confidence in the transformative potential of MPS and AI. History reminds us that revolutions, technological or otherwise, are rarely smooth transitions but rather periods of active debate, experimentation, and gradual acceptance. The ongoing adoption of animal-free biomedical methodologies is no exception, requiring persistent advocacy, education, and practical demonstration of effectiveness. We argued earlier that the most important ∼omics is economics (Meigs et al., 2018). Similar to more than a century ago (Fig. 4), it will be very much an economic discussion ultimately driving the transition, with the pharmaceutical industry likely in a lead role (Roth et al., 2021; Hartung, 2024a).

**Fig. 4:**
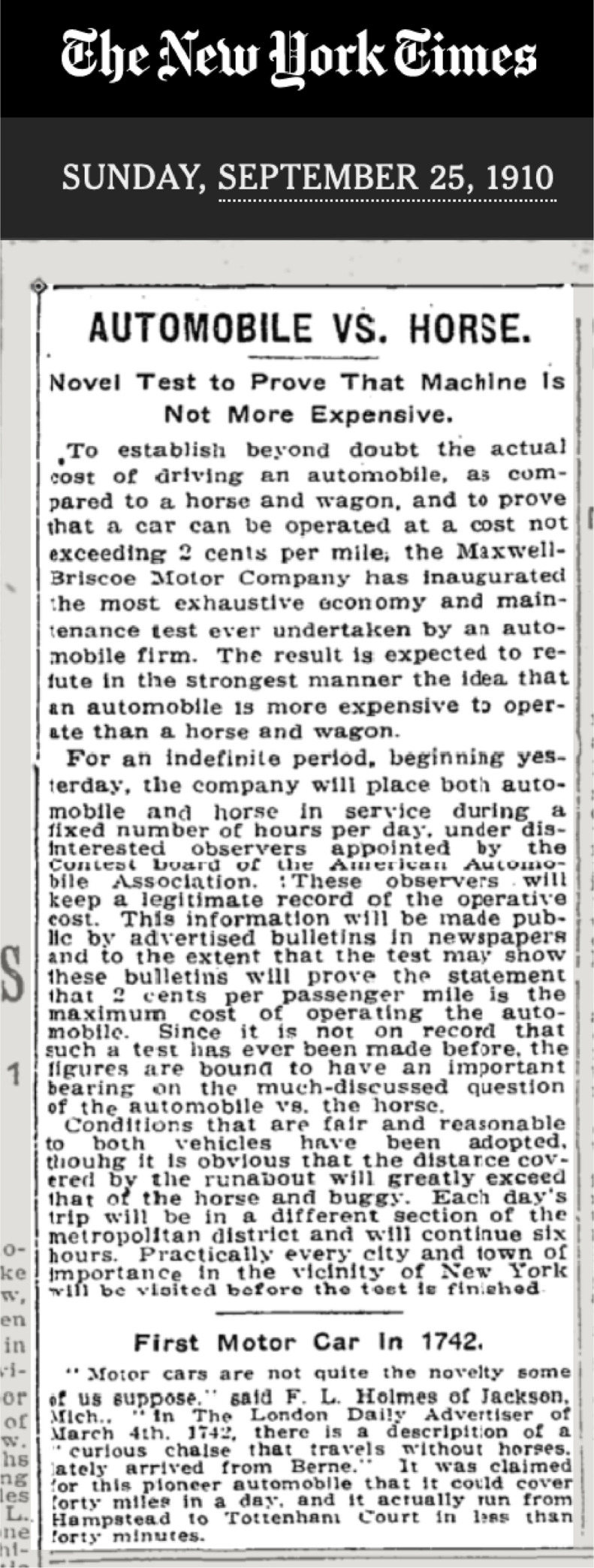
The economic argument.

Ultimately, the shift toward animal-free science represents more than technological progress; it symbolizes a profound ethical evolution paralleling society’s broader commitment to humane and scientifically advanced research methodologies. Just as automobiles became synonymous with modernity, mobility, and efficiency, animal-free technologies promise to define a new era of humane, precise, and ethically responsible biomedical research.

As we trade the familiar hooves for innovative chips, history’s humorous jeers and earnest skepticism remind us of humanity’s enduring apprehension towards radical change (Fig. 5). “The passing of the horse” has not yet found its match as “The passing of the lab rat”, but this might be only a matter of time. Yet, it also teaches us that embracing innovation eventually leads to significant societal advancements. Turning skeptics’ dismissive calls of “get a horse!" into pioneering declarations of progress requires vision, persistence, and collective courage—qualities abundant in today’s scientific pioneers.

**Fig. 5:**
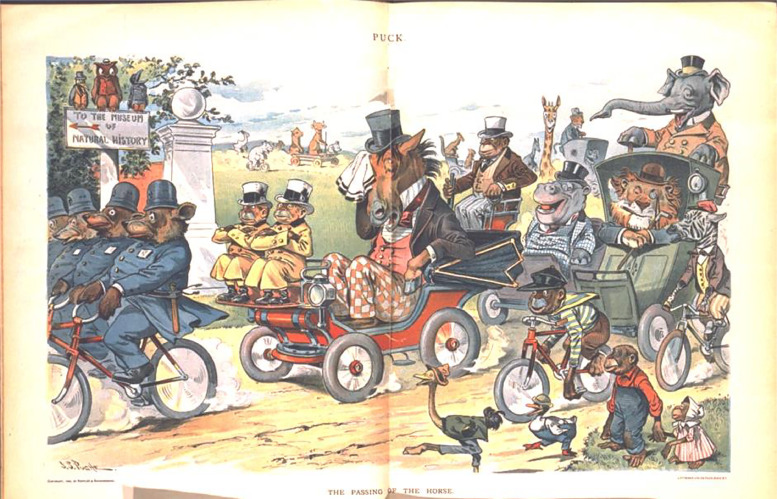
The passing of the horse by J.S. Pughe; J. Ottmann Lith. Co., N.Y.

Cartoon showing weeping horse in automobile with procession of other animals riding in automobiles and on bicycles; sign by road reads "To the Museum of Natural History." J. Ottman Lithographic Company. Pughe, J. S. (John S.), 1870–1909, artist. Published 1899 Feb. 22. Available at: https://www.loc.gov/item/00650802

## Declaration of interests

The authors declare that they have no known competing financial interests or personal relationships that could have appeared to influence the work reported in this paper.

## Data Availability

No data was used for the research described in the article.

## Footnotes

1 https://www.fda.gov/media/186092/download?attachment

2 https://www.washingtontimes.com/news/2025/apr/10/epa-chief-lee-zeldin-eyes-ban-animal-testing/
